# Independent aggregation in the nordic day-ahead market: What is the welfare impact of socializing supplier compensation payments?

**DOI:** 10.1016/j.heliyon.2024.e41619

**Published:** 2025-01-01

**Authors:** Kārlis Baltputnis, Tim Schittekatte, Zane Broka

**Affiliations:** a Institute of Power Engineering, Riga Technical University, Azenes iela 12/1, Riga, LV, 1048, Latvia; b MIT Energy Initiative, Massachusetts Institute of Technology, Cambridge, MA, 02142, USA; c Florence School of Regulation, European University Institute, Firenze, FI, 50133, Italy; d MIT Sloan School of Management, Massachusetts Institute of Technology, Cambridge, MA, 02142, USA

**Keywords:** Demand response, Electricity market, Compensation, Independent aggregator, Regulation, Market simulation

## Abstract

This paper addresses the participation of independent aggregators (IAs) for demand response (DR) in European electricity markets. An IA is an aggregator trading the flexibility of consumers of which it is not the electricity supplier. Particularly, we focus on the controversial issue of a compensation payment from the IA to the supplier for energy sourcing. Concretely, we explore the potential welfare impacts of partial or full socialization of the compensation payment. For this, a static simulation framework is introduced utilizing historical day-ahead market bid and offer data from the Nordic region to rerun market clearing under different scenarios of compensation rules and with diverse assumptions regarding the DR aggregated by IAs. The overall welfare impacts comprise of changes in producer and consumer surplus, DR consumer welfare impact and the socialized part of the compensation. Based on a case study utilizing data for the full year of 2018, it is found that subsidizing the participation of IAs in the day-ahead market leads to negative overall welfare impacts due to over-incentivization of demand reduction. However, in nearly all investigated cases the socialization of the compensation payment leads to positive impacts on the consumer surplus, driven by reduced electricity wholesale prices. The optimal share of the socialization of the compensation that leads to the highest net consumer benefit depends on many factors, among which the assumptions around activation costs of DR are the most evident. Nevertheless, we argue that the socialization of the supplier compensation should be at least conditional upon the level of the hourly wholesale price (i.e., a “threshold price”) and on DR cost estimates. Only in the case when high shares of untapped DR are relatively cheap, net welfare benefits will result.

## Highlights

• Welfare impact of demand response via independent aggregators is studied.
• Over-subsidizing independent aggregators can lead to overall welfare reduction.
• Consumer surplus is generally increased by supplier compensation socialization.
• There exists a sweet spot of socialization level, bringing the most consumer benefit.
• The optimal share of socialization depends on demand response activation costs.

## Abbreviations

DA: day-ahead
DR: demand response
EU: European Union
IA: independent aggregator
ICT: information and communication technology
MCP: market clearing price
MCV: market clearing volume
NEMO: nominated electricity market operator
RR: retail rate

## 1 Introduction

### 1.1 Background and motivation

#### 1.1.1 Demand response and aggregation

In order to keep the costs of the global energy transition under control, the power system needs to become more flexible [1]. Conventionally, this flexibility has been ensured by generation sources, such as reservoir or pumped storage hydropower plants and gas-fired turbines. The potential of demand-side flexibility, i.e. demand response (DR), has so far been relatively underexploited. According to Ref. [2], the theoretical European DR potential added up to about 100 GW in 2016, while only one fifth was actually active. This potential is expected to reach 160 GW in 2030. As extensively discussed in Ref. [3], in most European Member States, DR can be strengthened among industrial users and still needs to be opened to smaller grid users such as commercial and even household consumers. For example, Gils [4] concludes that around half of the potential for load curtailment can be found at the level of residential consumers. For load increase, this share grows to up to 80 %.

The DR potential of individual consumers is normally too low for direct participation in the electricity wholesale markets, capacity remuneration markets or ancillary service markets. This can be alleviated by pooling multiple flexible demand-side resources into an aggregated bid. In several countries, the aggregator function has been picked up by asset-light software companies that can equip flexible consumption devices with the ICT necessary for remote market-based activation of DR. One could think that the aggregator function would be undertaken by suppliers as they already have a close relationship with the consumers, but this has not necessarily been the case [5,6]. Reif et al. [7] discussed reasons such as the lack of incentive for suppliers and the need for specialized skills. They also state that by unbundling the supply and aggregation function, more competition in the aggregation business can develop.

In this regard, the role of independent aggregators (IAs) is deemed vital. IAs are entities that trade consumer DR while not being affiliated to the electricity supplier (i.e. retailer) of the respective consumers. IAs are formally recognized as new actors in the power system by the EU Clean Energy Package. The same legislation obliges Member States to devise rules allowing and fostering DR participation through aggregation in all electricity markets alongside producers in a non-discriminatory manner [8].

#### 1.1.2 IA implementation challenges

In practice, there are several difficulties in integrating DR and IAs in the existing electricity markets related to measurement and validation, baseline methodology, information exchange and confidentiality, transfer of energy price methodology, relationship between implicit and explicit DR, rebound effect and portfolio conditions [9]. One of the main concerns, if not the biggest current discussion around IAs, is whether they should compensate suppliers for the (potentially negative) financial effect caused to them due to DR activations by IAs. More precisely, when DR is provided through an IA, suppliers may be unable to invoice part of the energy they had purchased in expectation of the baseline load. Without any compensation mechanism in place, the IA is selling electricity it did not first buy, i.e. it is implicitly subsidized at the expense of the supplier. Alternatively, the supplier compensation can be socialized which equally benefits the business case for IAs but does not put a direct burden on suppliers. Article 17 of the Electricity Directive 2019/944 [8] leaves it up to the individual Member States whether to implement a supplier compensation mechanism and how exactly to design it, but it does point out that the socioeconomic impact of DR participation in the electricity markets may be considered:

“*Such financial compensation shall not create a barrier to market entry for market participants engaged in aggregation or a barrier to flexibility. In such cases, the financial compensation shall be strictly limited to covering the resulting costs incurred by the suppliers of participating customers or the suppliers' balance responsible parties during the activation of demand response. The method for calculating compensation may take account of the benefits brought about by the independent aggregators to other market participants and, where it does so, the aggregators or participating customers may be required to contribute to such compensation but only where and to the extent that the benefits to all suppliers, customers and their balance responsible parties do not exceed the direct costs incurred*.” [8].

There are several options for IA regulatory frameworks [[9], [10], [11], [12]], and their implementation models are summarized in Ref. [10]. An argument against implementing a compensation is it being a barrier to stimulating more DR. Increased DR could lead to cost savings and enhanced energy services for the final consumers resulting in an overall net benefit.^1^ For example, Chua-Liang and Kirschen [13] observe that increased DR participation leads to substantial savings in the power system operating cost that is transferred to the demand-side. Nevertheless, in most EU Member States with a framework already in place, a compensation mechanism has been introduced, but their implementation differs [10]. The two most important design choices are the level of compensation and the allocation of its costs. The Nordic countries, for instance, are striving for a harmonized approach. At the time of writing, there is some initial IA legislation in place for those reserve markets where main remuneration comes from the reserve capacity provided with relatively small activated energy amount. However, there are no rules yet clarifying how to handle compensation issues in markets with significant transfer of energy, incl. wholesale energy markets like the day-ahead (DA) market.

In 2020, the Nordic energy regulator association NordReg published an important position paper [14] advising against the “no compensation” approach as that would distort and harm the competitive and well-functioning electricity retail markets in the Nordics. Moreover, not being compensated for the actions of IAs could particularly harm small and recently entered suppliers, potentially causing their exit from the market and consequent reduction in supplier competition, whereas a high compensation might be seen as a barrier to IA emergence. On this basis, NordReg does point out that not all of the compensation amount necessarily must be paid by the IAs themselves, and it could be to some extent socialized through grid tariffs.^2^ Furthermore, NordReg admits that such socialization should definitely be subject to quantitative socioeconomic analysis [14]. However, there is not a universally agreed approach on how to perform it.

To develop their own IA regulatory framework, each Member State may require a thorough socioeconomic assessment of the alternatives, considering both the potential benefits from increased DR and the related costs. The results could then inform the methodology for introducing a specific compensation mechanism if this is deemed necessary. Solving this topic is left up to each Member State also, evidently, in the draft Network Code on Demand Response proposed by the ENTSO-E and the EU DSO Entity [15]. The lack of guidelines, among other reasons, has led to several EU countries lagging in full transposition of the Directive into the national legislation in particular concerning the DR participation via IAs in all electricity markets [16].

#### 1.1.3 Related work

The subject of DR impact on electricity market economic welfare has been mostly studied in the North American context, the liberalized markets of which were the first to enable DR bidding in practice. For instance, Walawalkar et al. uses an econometric model of locational marginal prices to simulate the wealth transfer from generators to consumers when DR is incentivized in the PJM market after the market price signals reach a certain “trigger point” [17]. They find that the subsidy can outweigh the welfare gains if the trigger is set too low.

Another relevant paper is the one by Chao et al. which use a conceptual framework and a simplified market model with free entry (i.e., wholesale prices unaffected by DR as they return to equilibria) to show how lack of compensation can induce inefficient underconsumption due to over-incentivization of DR [18]. Chao argues that by not having a compensation mechanism in place, i.e., paying out the full wholesale price to DR without a correction for the cost of the previously sourced baseline, double-payment incentives are created inducing excessive demand reduction resulting in net welfare losses. However, free entry is a strong assumption. It is well-know that power markets are not necessarily perfectly competitive [19]. In fact, one important benefit of increased DR is to mitigate market power [20]. Another key assumption in Ref. [18] is that all consumers are modeled to be price-sensitive and have the same elasticity. In reality, only a subset of consumers will be willing to be exposed to volatile price signals and be able to react to them.

In our study, we strive to expand this approach by Chao et al. by assuming only a part of consumption to be able to provide explicit DR via IAs, thus allowing us to test different assumptions regarding demand elasticity. To estimate how much wholesale prices are impacted by shifts in demand, we use historical aggregated bid and offer curves from the Nordic DA market published by its operator Nord Pool [21]. Using these curves, we simulate the impact of DR via IA on the overall welfare under different compensation schemes.

This DA market-focused approach is particularly relevant, as while DR can be valuable in both the system services markets and the wholesale energy markets, the latter have proven to be particularly challenging in terms of regulating the IA–supplier relationship without introducing barriers to DR [14].^3^ Importantly, this historical market curve-based approach allows us to test the opposite of the free-entry assumption from Ref. [18], i.e., have the wholesale prices be affected by DR and not return to previous price levels. No free entry is also a strong assumption, and we come back to its implication when discussing the results.

In the European context, there are a number of examples how the bid and offer curve data published by the DA market operators can be utilized in a static simulation framework to estimate the producer and consumer welfare impacts brought by various changes in the market situation. For example, a historical curve modification approach implicitly assuming no free entry has been used with Spanish DA market aggregated bid and offer curves to estimate the potential welfare and price impact of increased PV integration [22,23], wholesale price reductions achievable through energy efficiency and load shifting measures [24], and to compare the merit order effect obtainable by renewable generation versus demand-side management [25]. In Ref. [26], a similar approach has also been used the other way around, by removing renewable energy from the supply curve (instead of adding it) to estimate the value brought by renewables and contrasting it to the incentive payments they have received. While the outlined papers have studied some notable aspects of welfare impact, they did not consider the complex issues introduced more recently by the Electricity Directive such as the requirement to enable IA and DR participation in all electricity markets while regulating the compensation mechanism between IAs and suppliers.

Specifically Nordic DA market curves have been used for simulating the potential clearing price reductions achievable by increased amount of DR [27], but the implications of IA compensation mechanisms were not considered. Additionally, it was limited to socio-economic impact in terms of market price decrease without considering the broader welfare impacts. Altogether, the subject of IAs and especially their participation in wholesale markets has thus far received minor attention in research literature despite their clear topicality for policy-makers and potential impact on EU electricity markets.

### 1.2 Contribution

There is an evident research gap as, to the best of the authors’ knowledge, a thorough quantitative socioeconomic analysis of IA participation in European wholesale energy markets considering IA-supplier compensation issues has indeed not been addressed by the existing literature. This is primarily due to it being a comparatively recent topic and also due to, for the time being, more attention paid to integrating IAs in the ancillary services markets first [28,29]. Consequently, we provide two contributions to the body of knowledge.

1) A static simulation methodology to assess the welfare impacts of socializing IA-supplier compensation and the resulting net benefits which extends the existing literature [18] using tools previously applied in other contexts [[22], [23], [24], [25], [26]].
2) Analysis of the potential net benefits of integrating IAs in the Nordic DA market with varied degrees of compensation socialization.

In our paper, the aggregated market curve data enable simulating the activities of IAs in the DA market in several counterfactual scenarios, exploring the potential benefits obtainable and identifying possible over-incentivization risks. This is the first study quantifying conceivable IA–supplier compensation socialization scheme consequences in a European setting. Our work contributes to the ongoing European debate on how to unlock untapped DR in a just and economically efficient manner. Specifically, the employed methodology can evidently provide valuable insights as countries strive to devise approaches for assessing the socio-economic impacts of IA activities. Whereas the findings regarding socialization alternatives provide a broader understanding of ways of how to tackle the IA-supplier compensation.

The remainder of the paper is organized as follows. In Section 2, we explain the Nordic market context. In Section 3, we describe the methodology, including the metrics assessed and the simulation setup. In Section 4, we present the results and discuss the limitations of our approach. Last, we conclude, followed by appendices with additional results and sensitivity analysis.

## 2 The Nordic market context

The Nordic electricity wholesale market is regarded as one of the most successful power markets in the world [30]. Initially established in Norway in 1993 as a consequence of unbundling and liberalization, it gradually became a fully Nordic market by 2000 [31] and has also expanded elsewhere, fully integrated in the European single day-ahead market coupling.^4^ Cross-border trading enables the countries to benefit from utilizing their regional differences.

The DA market is the primary electricity trading platform in the region, and most of the power generation is marketed there. Nearly 90 % of the electricity consumed in the Nordics passes through the DA market, the remaining 10 % being traded by means of bilateral agreements [32]. The intraday market, consequently, acts as an adjustment market to the DA market with comparatively low trading volumes [33]. Compared to other European regions, only the Irish and Greek DA markets have higher churn factors, i.e., the volume of electricity traded over consumption, than the Nordic DA market. The DA churn factor is significantly lower in Central Western Europe (circa 20–40 %), highlighting major differences in how DA markets are utilized in various parts of the EU [34].

When discussing price and welfare impacts of DR, it is a simplification to use historical bid and offer curves instead of information about the marginal costs of producers and buyer willingness to pay. However, the high churn rate in the DA market allows us to justify the usage of historical aggregated bid and offer curves as a necessary approximation due to the lack of more precise information. Important in this regard is that the Nordic market has an even higher churn rate than the Iberian market (around 80 %) whose aggregated bid and offer curves have been used in previous studies [[22], [23], [24], [25], [26]].

## 3 Methodology

The basis of our methodology lies in modifying the actual bid and offer curves by the inclusion of IA offers, which are to a varied extent implicitly subsidized through IA-supplier compensation socialization. We run market clearing simulations for cases with and without these additional offers. We dub the latter (where IAs do not participate in the market) as the benchmark cases and the former (with IAs included under different compensation rules) as the alternatives. The impact of the compensation socialization alternatives is then estimated in the form of overall net benefit by comparing the sum welfare metrics of the alternatives versus the benchmarks.

Somewhat similar curve-based approach was already previously used to study the price-reducing effects of potential IA-sold load-reducing DR in the Nordic market [27]. However, in this study, instead of solely focusing on the wholesale price reduction, we assess overall welfare impacts, including also those caused by DR consumer over- and under-consumption [18]. To further extend the scope compared to Ref. [27], we also consider load-increasing DR in addition to load reduction. Moreover, in the revised methodology, instead of adding single-point DR bids, we assume flexible load marketed via IAs to be represented by multi-step DR activation curves.

In the following subsection we explain in detail how to calculate the overall net benefit using the outcomes of market clearing simulations of the benchmarks and the alternatives. Afterwards, in section 3.2 we explain the whole simulation procedure used in our case study on step-by-step basis.

### 3.1 Overall net benefit

In this study, the overall net benefit of IAs is calculated as the total welfare difference between the alternatives and the benchmarks. We have envisioned the possibility of having multiple benchmarks, since the DR activation cost curves are assumption based, and thus it is useful to be able to vary also this parameter. The welfare impact of DR (Eq. (1)) can be divided into four components as explained below: the difference in *producer surplus*, the difference in *gross consumer surplus*, the difference in *DR consumer welfare* and, finally, the *socialized* [part of the] *compensation payment*:

(1) $${ONB}_{b,s}=\sum_{t=1}^{T}\left({PS}_{t,b,s}^{\text{alt}}-{PS}_{t}^{\text{bm}}+{CS}_{t,b,s}^{\text{alt}}-{CS}_{t}^{\text{bm}}+{\Delta DRW}_{t,b,s}^{\text{alt}}-{\Delta DRW}_{t,b}^{\text{bm}}-k_{s}^{\text{comp}}\cdot RR\cdot {DRV}_{t,b,s}^{\text{traded}}\right)\;/\sum_{t=1}^{T}{MCV}_{t,b,s}^{\text{alt}}$$

where ${ONB}_{b,s}$ is the overall net benefit with each assumed DR cost curve *b* and socialization scenario *s*; ${PS}_{t,b,s}^{\text{alt}}$, ${PS}_{t}^{\text{bm}}$ – hourly *producer surplus* in the alternatives and benchmarks, respectively; ${CS}_{t,b,s}^{\text{alt}}$, ${CS}_{t}^{\text{bm}}$ – hourly gross *consumer surplus*; $\Delta {DRW}_{t,b,s}^{\text{alt}}$, ${\Delta DRW}_{t,b}^{\text{bm}}$ – hourly *DR consumer welfare* deviation from efficient consumption; RR – fixed retail rate; $k_{s}^{\text{comp}}$ – share of compensation socialized; ${DRV}_{t,b,s}^{\text{traded}}$ – volume of accepted (i.e., traded) DR in the alternatives*;* ${MCV}_{t,b,s}^{\text{alt}}$ – hourly market clearing volume (used to normalize the net benefit in €/MWh terms).

We can also focus solely on the consumer side benefits. For this, we remove the producer surplus components from Eq. (1) and calculate the consumer net benefit (${CNB}_{b,s}$). The components of ${ONB}_{b,s}$ and ${CNB}_{b,s}$ are obtained as follows.

#### 3.1.1 Change in producer surplus and gross consumer surplus

**Fig. 1:**
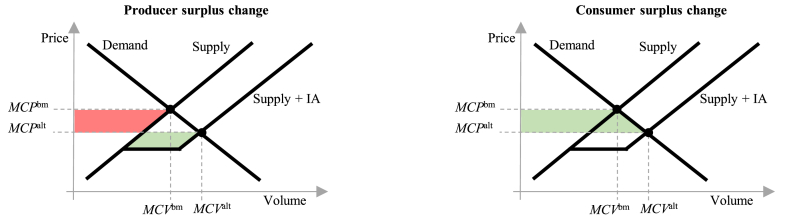
A stylized example of producer and consumer surplus changes due to addition of load-reducing DR offer on the supply side.

The change in *producer surplus* $\left({PS}_{t,b,s}^{\text{alt}}-{PS}_{t}^{\text{bm}}\right)$ and in gross *consumer surplus* $\left({CS}_{t,b,s}^{\text{alt}}-{CS}_{t}^{\text{bm}}\right)$ is calculated directly from the aggregated DA market bid and offer curves (the original curves for the benchmarks and the modified curves for the alternatives), representing the wholesale demand and supply respectively. In Fig. 1, a simplified situation is illustrated, whereby a single-step IA offer (load-reducing DR) is added to the offer curve as supply, shifting it to the right. In the figure, the red areas denote surplus reductions, but the green areas – increases. The formulae to calculate the producer and consumer surplus changes are given in Eq. (2) and Eq. (3), respectively. Do note that we make no assumptions on the actors whose bids construct these curves, and, in the alternative cases, the producer surplus does contain the surplus attributable to the IA load-reduction offers, whereas the consumer surplus includes IA load-increase offers.

(2) $${PS}_{t,b,s}^{\text{alt}}-{PS}_{t}^{\text{bm}}=\left({MCV}_{t,b,s}^{\text{alt}}\cdot {MCP}_{t,b,s}^{\text{alt}}-\int_{0}^{{MCV}_{t,b,s}^{\text{alt}}}P_{t,b,s}^{\mathrm{mod}.s}\left(V_{t,b,s}^{\mathrm{mod}.s}\right)\right)-\left({MCV}_{t}^{\text{bm}}\cdot {MCP}_{t}^{\text{bm}}-\int_{0}^{{MCV}_{t}^{\text{bm}}}P_{t}^{\text{orig}.s}\left(V_{t}^{\text{orig}.s}\right)\right)\text{,}$$

(3) $${CS}_{t,b,s}^{\text{alt}}-{CS}_{t}^{\text{bm}}=\left(\int_{0}^{{MCV}_{t,b,s}^{\text{alt}}}P_{t,b,s}^{\mathrm{mod}.d}\left(V_{t,b,s}^{\mathrm{mod}.d}\right)-MCV_{t,b,s}^{\mathrm{alt}}\cdot {MCP}_{t,b,s}^{\text{alt}}\right)-\left(\int_{0}^{{MCV}_{t}^{\text{bm}}}P_{t}^{\text{orig}.d}\left(V_{t}^{\text{orig}.d}\right)-{MCV}_{t}^{\text{bm}}\cdot {MCP}_{t}^{\text{bm}}\right)\text{,}$$

where ${MCP}_{t,b,s}^{\text{alt}}$ is the MCP in the alternative case at hour *t*, DR activation cost curve *b* and socialization scenario *s*; ${MCP}_{t}^{\text{bm}}$ – the hourly benchmark MCP; ${MCV}_{t}^{\text{bm}}$ – the benchmark market clearing volume; $P_{t}^{\text{orig}.s}\left(V_{t}^{\text{orig}.s}\right)$ and $P_{t}^{\text{orig}.d}\left(V_{t}^{\text{orig}.d}\right)$ – the original (benchmark) offer (supply) and bid (demand) curves; $P_{t,b,s}^{\mathrm{mod}.s}\left(V_{t,b,s}^{\mathrm{mod}.s}\right)$ and $P_{t,b,s}^{\mathrm{mod}.d}\left(V_{t,b,s}^{\mathrm{mod}.d}\right)$ – the modified (alternative) curves.

#### 3.1.2 Change in DR consumer welfare

**Fig. 2:**
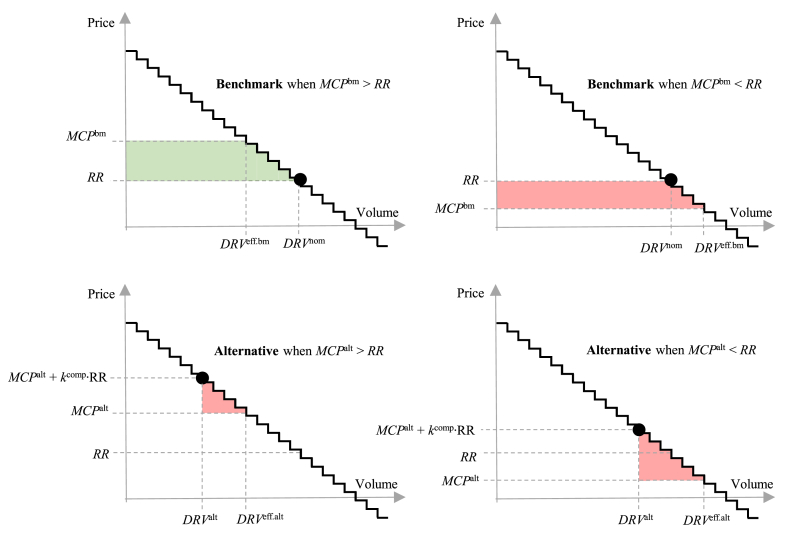
Visual explanation of DR consumer welfare impact estimation from the DR activation curve in the benchmarks and alternatives.

The change in *DR consumer welfare* from efficient consumption $\left({\Delta DRW}_{t,b,s}^{\text{alt}}-{\Delta DRW}_{t,b}^{\text{bm}}\right)$ allows estimating how trading DR via IAs in the DA market can impact the welfare of particularly those consumers who have some flexibility potential, especially in light of different supplier compensation mechanisms considered. These “correction terms” are necessary as the DR consumers aggregated by the IA are exposed to the market price plus the socialized part of the compensation. As explained before, the DR activation curves are assumption-based, thus we need to consider multiple cases with different flexibility characteristics. In the benchmarks, this assumed flexibility is not exposed to market price signals, instead being subject to a fixed volumetric rate. Thereby, as per [18], whenever the market price is higher than the fixed rate, the DR consumer is in fact over-consuming (i.e., consuming more than its efficient consumption), whereas when the market price is lower, the DR consumer is under-consuming. In the alternatives, under different shares of compensation socialization, the over-/under-consumption effects can be negated, or, on the contrary, consumption over-reduction can be incentivized, as shown in Ref. [18]. Fig. 2 illustrates the main principle behind the calculation of the ${\Delta DRW}_{t,b,s}^{\text{alt}}$ and ${\Delta DRW}_{t,b}^{\text{bm}}$ values. The green area indicates surplus increase in terms of the DR curve and the red areas stand for a reduction. Eqs. (4), (5) show the mathematical approach in calculating the respective surplus changes.

(4) $${\Delta DRW}_{t,b}^{\text{bm}}={MCP}_{t}^{\text{bm}}\cdot {DRV}_{t,b}^{\text{eff}.\text{bm}}-RR\cdot {DRV}_{t,b}^{\text{nom}}-\int_{{DRV}_{t,b}^{\text{nom}}}^{{DRV}_{t,b}^{\text{eff}.\text{bm}}}{DRP}_{t,b}\left({DRV}_{t,b}\right)\text{,}$$

(5) $${\Delta DRW}_{t,b,s}^{\text{alt}}={MCP}_{t,b,s}^{\text{alt}}\cdot \left({DRV}_{t,b,s}^{\text{eff}.\text{alt}}-{DRV}_{t,b,s}^{\text{alt}}\right)-\int_{{DRV}_{t,b,s}^{\text{alt}}}^{{DRV}_{t,b}^{\text{eff}.\text{alt}}}{DRP}_{t,b}\left({DRV}_{t,b}\right)\text{,}$$

where ${DRV}_{t,b}^{\text{eff}.\text{bm}}$ is the efficient DR consumption at the benchmark MCP; ${DRV}_{t,b}^{\text{nom}}$ – the normal DR consumption at *RR* (i.e., when DR is not traded in the DA market via IAs); ${DRV}_{t,b}^{\text{eff}.\text{alt}}$ – the efficient DR consumption at the alternative MCP; ${DRV}_{t,b,s}^{\text{alt}}$ – modeled DR consumption in the alternative; ${DRP}_{t,b}\left({DRV}_{t,b}\right)$ – the assumed DR activation cost curve *b*. Eqs. (4), (5) are suitable for a generalized case where there can be a different DR activation curve for each time unit. However, in this study, the curves are not assumed to be time-dependent, thereby index *t* can be removed from ${DRP}_{t,b}\left({DRV}_{t,b}\right)$ and ${DRV}_{t,b}^{\text{nom}}$. For the latter, the index *b* can be discarded as well, since the consumption without market exposure should be equal regardless of the assumed cost curve, as this DR does not have access to the market via the IAs in the benchmarks.

#### 3.1.3 Socialized compensation payment

Finally, the last component from the overall net welfare impact (${ONB}_{b,s}$, Eq. (1)) and consumer net welfare impact (${CNB}_{b,s}$) calculation that remains to be explained is the *socialized compensation payment* $k_{s}^{\text{comp}}∙RR∙{DRV}_{t,b,s}^{\text{traded}}$. The volume of the successfully traded DR energy is related to the DR consumption in the alternatives:

(5) $${DRV}_{t,b,s}^{\text{traded}}={DRV}_{t,b}^{\text{nom}}-{DRV}_{t,b,s}^{\text{alt}}\text{.}$$

Consequently, in case of load-reducing DR, ${DRV}_{t,b,s}^{\text{traded}}$ is positive and the socialized compensation component in Eq. (1) is negative, whereas in case of load-increasing DR, ${DRV}_{t,b,s}^{\text{traded}}$ is negative and the socialized compensation has a positive sign.^5^ I.e., in the latter case, the suppliers share all or part of their extra revenue from increased DR load with all consumers.

### 3.2 Simulation data and procedure

We employ the priorly described methods and equations for overall net benefit and consumer net benefit calculation on a case study based on the data from the Nordic DA market operator Nord Pool. Our case study is based on data from a full year of 2018. For an explanation of this selection and an overview of the data analyzed, we refer to Appendix A. The quality of the data published by the market operator for the year 2018 was assessed by Baltputnis and Broka in Ref. [27], where it was found that they are sufficient for correct replication of the actual system price for 88.4 % hours. In only 1.4 % of the hours the error exceeded 0.02 €/MWh (which could be explained by accumulated rounding issues), and only for 0.2 % they exceeded 0.50 €/MWh. For hours with error >0.02 €/MWh, the original price was 24.15–66.90 €/MWh with an average of 41.23 €/MWh (i.e., not outliers), thus signifying that the data is of sufficient quality for use in this study.

**Fig. 3:**
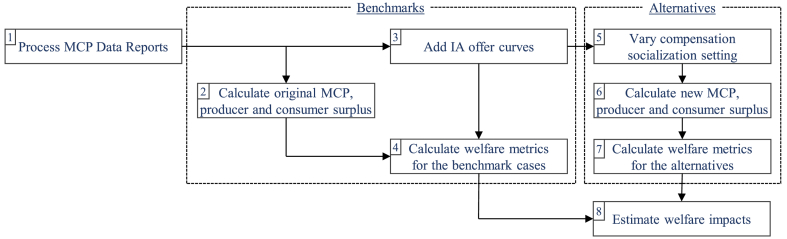
The overall setup of the day-ahead electricity market simulations to estimate the welfare impact of DR via IAs.

**Fig. 4:**
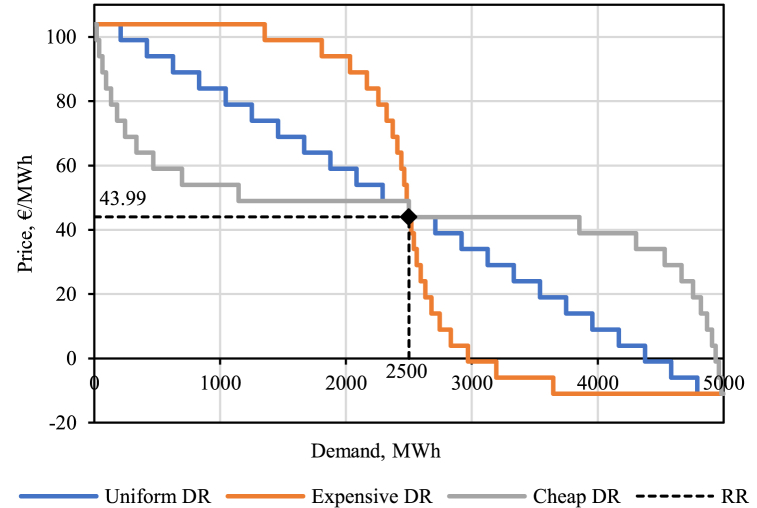
The assumed DR activation cost curves.

The simulation process for our case study is explained visually in Fig. 3. Its main steps are as follows:

1) Download the market clearing price (MCP) data reports from Nord Pool website [21]. Each file contains the aggregated bid and offer curve data for a single day. These data need to be further processed to enable reconstruction of the original MCP and modification of bids. This involves separating the buy and sell volume and price data points for each day and adding the respective accepted block bid volumes and net flows with neighboring areas to each point of the respective curve (as explained in Ref. [35]).^6^
2) Once the curves are reconstructed from the source data, their intersection point can be identified, thereby finding the original MCP. While there are a number of methods to calculate the cross point between two curves, in this study, we use the Fast and Robust Curve Intersection algorithm [36]. The respective benchmark surpluses (right side brackets from Eqs. (2), (3)), on the other hand, are obtained with trapezoidal integration, since in practice the bid and offer curves are given as discreet variables (price–quantity pairs).
3) Next, assumptions are made regarding DR potential which could be brought to the market by IAs. In this study, we assume three distinct DR activation curves as outlined in Fig. 4. They have the same price steps with increments of 5 €/MWh and the nominal (baseline) consumption of 2500 MWh^7^ at a fixed retail rate (*RR*) of 43.99 €/MWh (as a simplification, the assumed *RR* is equal to the average actual DA price in 2018). We assume the DR consumers have an inherent flexibility to reduce their consumption to 0 at a price of 103.99 €/MWh and, conversely, double the consumption at prices below −11.01 €/MWh. Each curve has 24 steps. What distinguishes the three curves are the volume changes per 5 €/MWh step. For the *Uniform DR* (blue in Fig. 4) the volume increments are constant; for the *Expensive DR* (orange) they are the smallest near the nominal consumption and increase farther out; whereas for the *Cheap DR* (grey) it is the other way around with the largest volume changes near the nominal consumption. The different curves provide a sensitivity with regard to the DR activation cost curves, which will be a function of the context and for which we lack empirical estimates. It should be stressed that by utilizing this approach we do not add any additional load to the Nordic system when MCPs remain the same, i.e., in the benchmark cases, load flexibility is untapped, while, in the alternatives, it is traded in the DA market via IAs (“load reduction/increase”). Thereby total load volumes can change whenever DR bids impact the MCP. We do not consider intertemporal links for the flexible demand (“load shifting”). This is a potentially valuable extension of the model that can be explored in future work.
4) Depending on the DR activation curve, for each hour a welfare impact can be calculated from not subjecting the flexible load to the wholesale prices but instead to the flat retail rate. We estimate these welfare impacts to calculate the benchmark welfare for each DR activation curve in accordance with Eq. (4).
5) We expose the DR activation curves to the DA market via IAs, with varying share of IA–supplier compensation being socialized ranging from 0 % to 100 % with an increment of 5 percentage points. We call these 21 scenarios per DR curve the “alternatives”. The compensation payment from the IA to the supplier can be formalized as in Eq. (6):

(6) $${Compensation}_{t,b,s}=-\left({1-k}_{s}^{\text{comp}}\right)\cdot RR\cdot {DRV}_{t,b,s}^{\text{traded}}$$

**Fig. 5:**
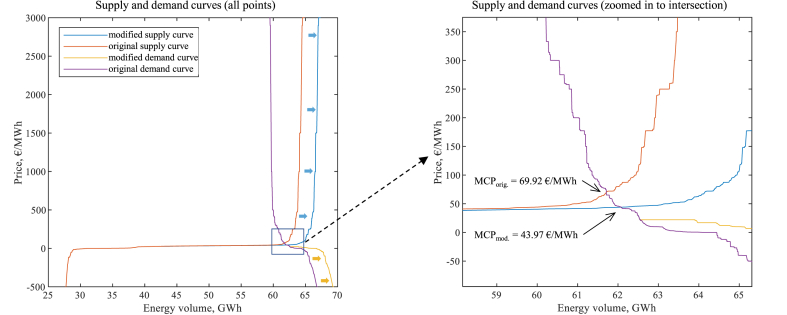
Example of MCP change due to market curve modification (based on 2018.02.26 07–08 CET data and *Cheap DR* curve at 50 % compensation socialization).

In case the compensation is negative (under load-increasing DR and a degree of socialization lower than 100 %), the supplier pays the IA instead. The socialized part of the compensation is allocated to all consumers. As explained in the next step, the level of compensation paid by/to the IA will directly impact its bidding strategy.

6) The curves from Fig. 4 are deconstructed into load-reducing DR and load-increasing DR components and added to the respective market curves (i.e., aggregated supply and demand). An example of this is shown in Fig. 5. The price steps are modified in accordance with the compensation alternative under study (e.g., with full compensation (no socialization) the first increment of load-reducing DR is sold at 5 + 43.99 €/MWh, whereas with no compensation (full socialization) it is sold at 5 €/MWh). Due to the addition of new bids at certain price points, portions of the original curves are shifted to the right. In other words, the DR activation curves are exposed to the MCP plus the socialized part of the compensation payment. Once the new MCP is obtained, we calculate also the producer and consumer surpluses for the alternatives (i.e., in accordance with the left brackets of Eqs. (2), (3)).
7) In the next step, the over-consumption (or under-consumption) surpluses of the three DR cost curves are calculated for the alternative cases, according to Eq. (5). Importantly, in the alternative cases, the DR activation curve is not subject to the MCP but the MCP plus the socialized part of the compensation. It can be expected that the more socialized the compensation payments are, the more over-incentivized load-reducing DR becomes (compared to when that DR load would be subject solely to the MCP), whereas load-increasing DR turns increasingly less attractive. Afterwards, the welfare metrics of interest are calculated for the alternatives.
8) Finally, the metrics obtained for the alternatives can be contrasted to the benchmark cases to estimate the welfare impacts and the overall net benefit (Eq. (1)) and net consumer benefit of the varied compensation socialization alternatives for the three DR activation cost curves.

Note that these calculations regarding the DA market clearing (a day before delivery) are not affected by uncertainty related to DR activation in delivery time. This is because it concerns a different timeframe and in case of non-delivery the IA would be subject to imbalance settlement, hence incentivizing it to schedule and ensure the activations appropriately.

## 4 Results

### 4.1 Overall net benefit

**Fig. 6:**
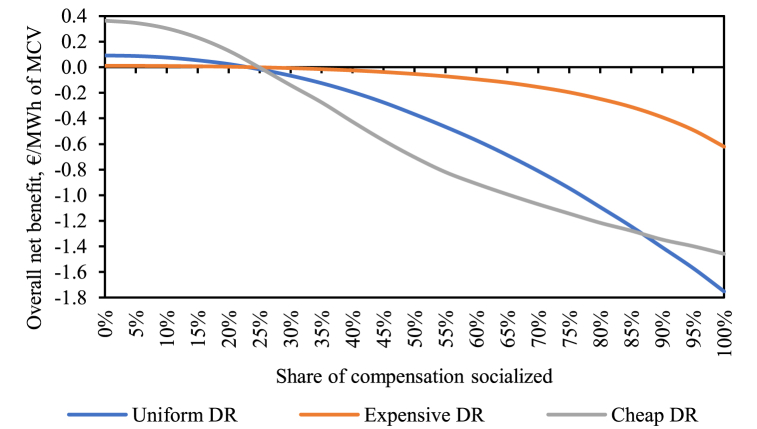
Overall net benefit divided by the total clearing volume with the assumed DR activation curves and varied shares of socialization of the compensation.

The key results are shown in Fig. 6. It displays the overall (static) net welfare benefit per DR activation curve expressed per unit of the total traded energy during the entire year, i.e., as expected values relative to market clearing volume (MCV), for different compensation socialization alternatives. We make three observations. First, for our case study, the benefit is highest without any socialized compensation for all three DR activation curves. The more of the compensation is socialized, the lower is the net welfare benefit. Second, at about 25 % of socialization the net benefit of the introduced DR changes from being positive to negative, independently of the DR activation curve. Third, the net benefit curves are shown to react non-linearly to socialization and to be sensitive to the DR activation cost curves. The cheapest DR curve can provide the highest net benefit (0.36 €/MWh with no compensation socialization in place), but it quickly becomes the most harmful at socialization higher than 25 %. Only at a very high socialization (above 90 %) the net benefit is most negative for the medium DR activation cost curve (up to −1.75 €/MWh under full socialization). The *Expensive DR* activation cost curve seems to be the least sensitive to the socialization alternatives with the positive net benefit already very low under no socialization and the welfare reduction being the most limited under high shares of socialization. In what follows, we discuss in more depth the decomposition of the welfare changes into the four components as described in Eq. (2) and discuss the important drivers behind the results.

### 4.2 The different components of net benefit

**Fig. 7:**
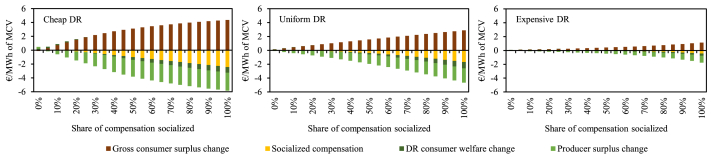
Welfare components under the different DR activation cost curves and compensation socialization alternatives.

Fig. 7 shows the overall net benefit components under the different DR activation cost curves and varied compensation socialization. As expected, the impacts on the different individual welfare components are highest under the cheapest DR cost activation curve. We discuss each component one by one.

First, the producer surplus. As, due to the implementation of the socialization mechanism, the reduction of consumption is additionally incentivized, while the increase of consumption is disincentivized, MCPs will decrease with more socialization. As market prices decrease, change in producer surplus is overwhelmingly negative with higher shares of socialization. Only in the case of *Cheap DR* and no socialization, the producer surplus change is positive (+0.27 €/MWh) and, consequently, the change in gross consumer surplus negative (−0.12 €/MWh). This is due to stronger average impact of load-increasing DR activations which raise instead of reducing the MCP as would be the case with dominantly load-reducing DR activations.

Second, conversely, the gross consumer surplus change is always positive except for the case of *Cheap DR* and no socialization. Overall, decreasing market prices lead to increases of the gross consumer surplus. Interestingly, the positive impact on the gross consumer surplus is greater than the negative impact on producer surplus as long as we do not consider the other two consumer surplus components.

Third, we explore the DR consumer welfare change. Important to notice that this component is measured relative to the benchmark, i.e., a fixed retail rate and thus no changes in the consumption patterns. Under the no socialization alternative, consumption is behaving optimally efficiently as it is being subjected to the wholesale price (thus no distortion). Thereby, this additional DR consumer welfare change is positive if there is more under-than over-consumption in the benchmark case. With higher shares of socialization, the distortion increases, and the DR consumer welfare change becomes more negative as we increase the incentive to under-consume. In our results, we see that for all DR activation curves, this component is the highest at no socialization and then decreases. It remains (marginally) positive for degrees of socialization up to about 25 % as in those cases the consumption is generally rather increased than decreased. This component becomes increasingly negative when the socialization coefficient is higher. Compared to the other components, it provides on average the least impact on the overall net benefit as we only assume a relatively small part of demand being contracted by IAs unlocking its flexibility. However, in individual instances the weight of this component can be significantly more pronounced; see Appendix B for an example based on the hour with the highest original market price.

Fourth, the socialized compensation component is generally negative and increases in magnitude with higher shares of socialization of the supplier compensation. The only exception is the case of 5 % socialization where the compensation is marginally positive for all DR activation curves. This can be explained by the relatively high volume of load-increasing DR which offsets the socialized compensation costs.

In general, we can conclude that higher shares of socialization of the supplier compensation lead to increased transfer from producer surplus to gross consumer surplus. At the same time, with higher shares of socialization, the socialization costs increase, and the DR consumer surplus turns more negative. The largest consumer net benefit does not necessarily occur at a 100 % compensation socialization. We investigate the consumer net benefit in more detail in the next subsection.

### 4.3 Consumer net benefit

**Fig. 8:**
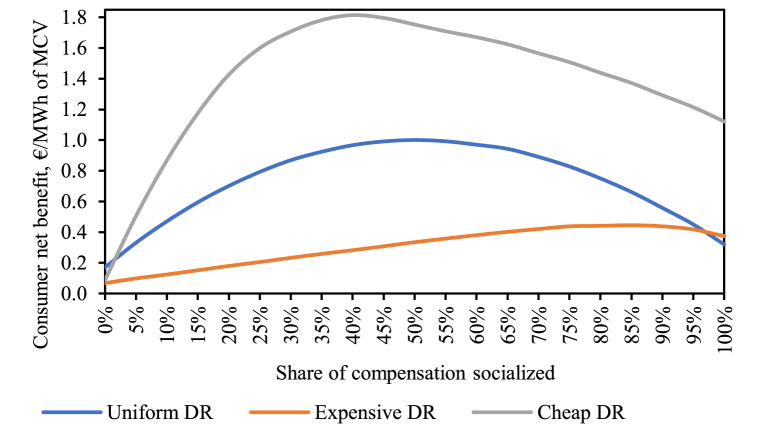
Consumer net benefit under the different DR activation cost curves and varied socialization alternatives.

Consumer net benefit, the sum of the three consumer-related components from Fig. 7, is shown in Fig. 8. We make again three observations. First, the expected consumer net benefit is always positive in our case study regardless of the socialization alternative. Moreover, the cheaper DR is, the more an average consumer gains. Second, evidently, there exists an optimal share of socialization with respect to the consumer net benefit, but that optimum is DR activation curve specific. Intuitively, the cheaper DR, the lower the optimum share of socialization. Indeed, the maximum consumer net benefit is achieved at 40 % of socialization for the *Cheap DR* (1.81 €/MWh), at 50 % for the *Uniform DR case* (1.00 €/MWh) and at 85 % for the *Expensive DR* (0.45 €/MWh). Third, the consumer net benefit is the lowest at no socialization in contrast to the overall net benefit metric, which showed the greatest value at 0 %.

**Fig. 9:**
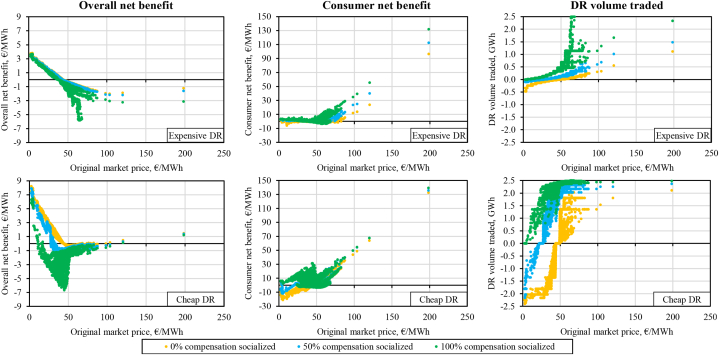
Overall net benefit (*left*), consumer net benefit (*center*) and DR volume traded (*right*) each hour depending on the original market price for the *Expensive DR* and *Cheap DR* activation curves.

The reductions in the consumer net benefit when the socialization share is increased beyond the optimal point for each activation cost curve expose a cannibalization effect. Evidently, increasing the socialization incentivizes more IA activity of load reduction at market prices around the average price (43.99 €/MWh) or below. This can be seen on the right-side charts in Fig. 9 for the *Cheap DR* and *Expensive DR* activation curves. These figures showcase the hourly overall welfare, hourly net consumer welfare and hourly activated DR in the modeled year for three selected socialization alternatives (0 %, 50 % and 100 %). The data points are sorted according to the original market price.

Fig. 9 reveals the dynamics behind the shape of the consumer net benefit curve. With increasing socialization up to the optimum point, the consumer net benefit grows because of two underlying drivers. One of the drivers is disincentivization of load-increasing DR actions which tend to increase the market price. This is clearly shown on the right-side graphs where load-increasing DR (negative volume in the charts) is most active at no socialization, significantly less active at 50 % and non-existent at full socialization. Another driver is the additional incentive to also reduce higher volumes of load at hours beyond the level that would be profitable if no socialization of the compensation would be in place. This can be seen in the middle graphs of Fig. 9. For example, for *Expensive DR* between the original market price levels of 50 and 100 €/MWh the consumer net benefit is significantly higher with higher socialization. Beyond the optimum point for the consumer net benefit, the costs of socialization and DR consumer welfare reduction start to counteract more strongly the improvements in gross consumer surplus. This is particularly evident in the middle bottom graph for *Cheap DR*: around original price levels at about ±20 €/MWh of the average original DA price (43.99 €/MWh) a significant share of DR load-reduction actions actually results in a decrease of the consumer net benefit, which is significant since about 96.19 % of all data points fall within this range for the studied year. This is less pronounced in the case of *Expensive DR*.

**Fig. 10:**
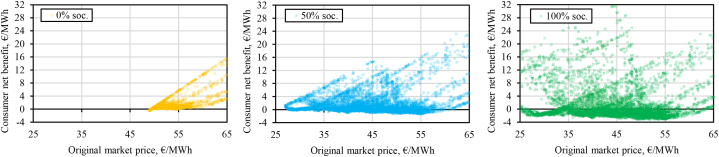
Hourly consumer net benefit vs original market price within a selected MCP range (25–65 €/MWh) focusing only on load-reducing DR events (with *Cheap DR* activation cost curve and three compensation socialization alternatives).

This effect is more discernible if we only focus on the hours with load-reducing DR and zoom in to the said range (original market price of 25–65 €/MWh) as shown with the *Cheap DR* example in Fig. 10. Comparing the 50 % and 100 % socialization alternatives, there are more positive impact instances in the full socialization alternative. However, the rise of negative impact events is significantly more pronounced. While each negative effect is comparatively minor in terms of magnitude, their sheer number pushes the average consumer net benefit towards a smaller value than in the 50 % socialization case. Combined with a decreased producer surplus, load-reducing DR at full compensation socialization leads to the most negative overall net benefit as can be seen in the left-side graphs in Fig. 9.

Curiously, however, with *Expensive DR* the overall net benefit is negative even at high original market prices irrespective of the socialization alternative. This can be explained by DR consumer welfare reduction, whereby their benchmark over-consumption at high prices is negated when exposed to market signals. This is addressed in more detail in Appendix B, whereas Appendix C deals with the sensitivity of the results on DR volume and cost assumptions, wherein it is found that under some very specific circumstances high compensation socialization can lead to also the consumer net benefit being negative.

Overall, however, we can see that, looking strictly from the consumer perspective, to some extent incentivizing (through implicit subsidies via compensation socialization) IA activities can bring benefits to the consumers at the cost of producers. While it could be argued that such a market distortion through subsidized DR-reduced market prices might send wrong price signals to investors in power production, it could, on the other hand, act as a factor remedying imperfections on the supply side, such as major actors imposing market power.

### 4.4 The business case of the IA

**Fig. 11:**
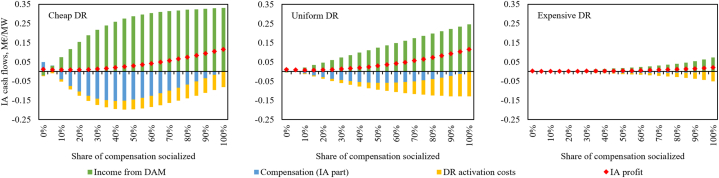
IA activity cash flows per DR capacity with the assumed DR activation cost curves and varied compensation socialization.

The cash-flows related to IA activities in the DA market can be expressed by three main components: (1) income from the DA market, (2) IA part of the compensation payment and (3) DR activation costs. Let us express the profitability in the studied year on the basis of DR capacity (2.5 GW according to the assumptions); note that we do not consider the capital and fixed operational costs of IA business. The results are shown in Fig. 11. As could be expected, the more expensive DR is, the less profitability it offers to the IA. Such DR is more expensive (per unit of energy) to begin with and therefore activated less often, thus generating lesser profit.

Despite the highest overall net benefit from IA activities in the DA market achievable with no compensation socialization (Fig. 6), such a scenario is very unlikely to promote IA emergence as it provides the least opportunities for IAs to generate profit. IAs have a very low expected annual profitability per MW of load-reducing DR bid with no or low compensation socialization. On the other hand, load-increasing DR provides a higher value to the IAs even without compensation. However, compared to any alternatives with some socialization, the IA income from load-increasing DR is minuscule compared to load reduction. Another important takeaway is that IA profitability keeps gradually increasing with rising compensation socialization, despite providing progressively more negative overall net benefit (Fig. 6) and reducing consumer net benefit towards high socialization shares (Fig. 7). Indeed, IA profitability grows with increased socialization even if the benefits to consumers start to diminish. It should be noted, though, that Fig. 11 assumes full DR availability on the DA market at the maximum capacity each hour, likely significantly overestimating the cash-flow per MW of DR capacity as usually an IA manages a larger portfolio of diverse DR assets with a larger total capacity than offered on the market each hour.

It could be reasoned that DR could be rather employed within the DA market framework implicitly via dynamic tariffs (based on market prices), which are in fact quite common in the Nordics already. Moreover, comparable price-reducing benefits could just as well be created by subsidizing the creation of new power plants (especially renewable energy based). However, as mentioned in Section 1, the Clean Energy Package calls for non-discriminatory participation and barrier-free entry of IAs in all markets, including the DA. Hence, it could be also argued that being subject to full supplier compensation almost eliminates any business case for the IA and effectively bars them from entering and operating in the DA market.

### 4.5 Key assumptions and simplifications

The methodology employed for this study is subject to a number of major assumptions and simplifications necessary, first, to ensure that the simulations are tractable, second, to limit the scope of the study isolating the particular topic of interest and, third, to respect limitations imposed by the available data. Apart from those mentioned in the prior subsections, a number of other points need to be addressed. Consequently, the results and conclusions presented in this paper must be viewed in light of the outlined limitations.

A key assumption is the notion that the modeled DA price changes due to DR traded by IAs would not be corrected by free entry. Essentially, the model considers that additional entries by IAs in the bid and offer curves are the only modification of these historical curves and the bidding behavior of other market actors remains unaffected. Consequently, such static analysis is best suitable to estimate the short-term effects [22], since in the longer term, the market participants would likely adapt to the evolving market situation. E.g., reduced producer surpluses might lead to less investment in production assets, which, in time, might negatively impact the consumers due to rising electricity prices. Additionally, it might lead the large-scale Nordic hydroelectric power producers to change their strategic bidding behavior to try and counteract their surplus losses, using their flexibility to take up some of the IA market share. Disregarding these possibilities leads to our simulated IA activities and their resulting impacts to be potentially overestimated.

Since the static analysis presented in this paper exploits the actual historical market data, the counterfactual scenarios studied operate under what-if assumptions on fully fledged IAs being present in the DA market of the region already in the studied time period. It follows, the model disregards potential changes in the future generation mix and other evolving medium and long-term factors, including those that do not depend on IA actions, such as the expected rise of intermittent renewable energy generation capacities. This could induce increasingly variable market prices, potentially leading to greater IA activity, unlocking more consumer benefits and profitability for IAs. However, this approach is in line with the objectives of the study which aim to benefit from the historical data availability.

Another major but necessary assumption is that the IAs are implied to know the compensation price in advance and, consequently, can properly price it into their bids. This allows to fully examine the impacts of socialization-induced over-incentivization of IAs, since they are not subjected to uncertainty (and thus increased risk-aversion) regarding *ex-post* calculation of the compensation payment. Nevertheless, such an *ex-post* approach cannot be entirely dismissed and conceivably could be addressed in future work by a modified version of the methodology employed in this study. However, the implied reduced uncertainty in the present study likely leads to overestimation of IA activities. Additionally, we disregard network tariff, tax and levy induced impacts on DR behavior, focusing solely on the energy component of electricity payments, which might also lead to some overestimation.

The final point to note is that Nord Pool publishes the anonymized bid and offer curve data aggregated for the Nordic region (Denmark, Finland, Norway, Sweden), thereby they cannot be used to calculate the prices in individual bidding zones. Instead, it enables calculating the Nordic system price, which is the unconstrained market clearing reference price for the region, intended to ensure a common benchmark for the Nordic DA market [37]. In general, the system price tends to vary less than area prices. Because of this, some underestimation of IA activities and cash-flows could be expected in contrast to other simplifications. This is also the case if we consider a potential future situation with larger price volatility because of increased share of intermittent renewable generation, such as wind, as a result of which the need for more DR will likely increase. Moreover, in the years following the selected study year of 2018 the market situation in the Nordic region has changed notably. E.g., there were significantly more hours with comparatively high prices especially during the European energy crisis in 2021–2022. As discussed, this would be likely beneficial for the IA business case. Nevertheless, it is important to note that a practical use of our assessment approach would not be hindered by bidding zone and multi-NEMO data availability issues, as the prospective users of it, especially regulators, would have sufficient access to the actual disaggregated market curve data, whereas we relied entirely on openly available data.

An additional important simplification is that we looked at strictly DR-aggregating IAs. While IAs could also include generation assets, this was not a subject of this study and thus not considered (as the supplier compensation issue is not relevant for generators). The DR cost curves assumed could also be implied to have some behind-the-meter storage; we did not elaborate on this as we are agnostic on the nature of DR assets behind our assumed simplistic multi-step curves. Likewise, we do not expect the IAs to exhibit strategic bidding behavior and instead assume they offer their flexibility to the market at cost, considering also the unsocialized compensation. This is a rational expectation for a DR-only trader in a market as large as the Nordics, especially as we do not consider the IAs’ portfolios to contain other types of assets. The DR curves were selected to cover a range of generalized options. However, since the welfare metric calculation depends on these curves (especially the DR consumer welfare impact), they are a factor somewhat limiting the practical usability of the utilized net benefit estimation method, whereby the DR curves would have to be constructed on a wide range of assumptions.

## 5 Conclusions

In this paper, we used aggregated market data on the bid and offer curves of the Nordic market operator Nord Pool within a static simulation framework to evaluate the potential welfare impact of IA entry in the DA market and estimate the consequences of supplier compensation socialization. While IAs can be active in all electricity markets, we focus here on the DA wholesale market as the volume of transferred energy by IA active in this market relative to other markets is expected to be the highest, and hence the question around compensation is most pressing, especially if the compensation price is expected to be in some way tied to the DA market price. The main finding is that while a partial socialization of the IA's payment to the supplier can lead to an optimal net consumer benefit in the short-run and strongly benefits the business case of IAs, the overall welfare impact of such a compensation rather quickly turns negative (in our case study at 25 % of socialization independent of the assumptions on DR activation costs). The main mechanism behind this is the major reduction in producer surplus due to the decreased wholesale price. This is especially due to demand reduction occurring near average price levels, incentivized by the high share of the compensation payment socialized.

Considering these findings, while also acknowledging the potential overall socioeconomic benefits of the emergence of IAs (e.g., unlocking “dormant” DR and decreasing market power), a regulatory compromise for policy-maker consideration could be a partial socialization of the compensation payment above a certain price level (e.g., twice the average wholesale price). We have seen from our results that such activations do limited harm to the overall welfare while it would stimulate the IAs business case. Such a rule would be similar as the net benefits test of DR introduced in the United States, even though implementation needs to be considered carefully. Considering the recent European energy crisis and future greater price volatility as the penetration of intermittent renewables continues, the importance of DR is expected to increase and hence an adequate regulatory framework is vital. To that end, our proposed socioeconomic analysis approach can be employed to inform certain aspects of the IA regulatory framework, especially in the EU countries transposing the Electricity Directive.

The results presented in this paper are based on the historical market data, which has certain limitations. When considering the specific findings, they must be viewed in the context of the methodology and the underlying data which for this study was based on the year 2018. Arguably, the most important simplification is that of price changes induced by IA activities not being corrected by free entry (e.g., it is ultimately a static short-term assessment).

## CRediT authorship contribution statement

**Kārlis Baltputnis:** Writing – original draft, Visualization, Software, Methodology, Funding acquisition, Formal analysis, Data curation, Conceptualization. **Tim Schittekatte:** Writing – original draft, Validation, Methodology, Investigation, Formal analysis, Conceptualization. **Zane Broka:** Writing – review & editing, Visualization, Validation, Investigation, Conceptualization.

## Data availability

The code devised to obtain and process the day-ahead market curve data and perform the simulations is available on GitHub: https://github.com/Karlis-Baltputnis/independent-aggregator-day-ahead-market.

## Declaration of competing interest

The authors declare that they have no known competing financial interests or personal relationships that could have appeared to influence the work reported in this paper.

## Figures and Tables

**Fig. A1:**
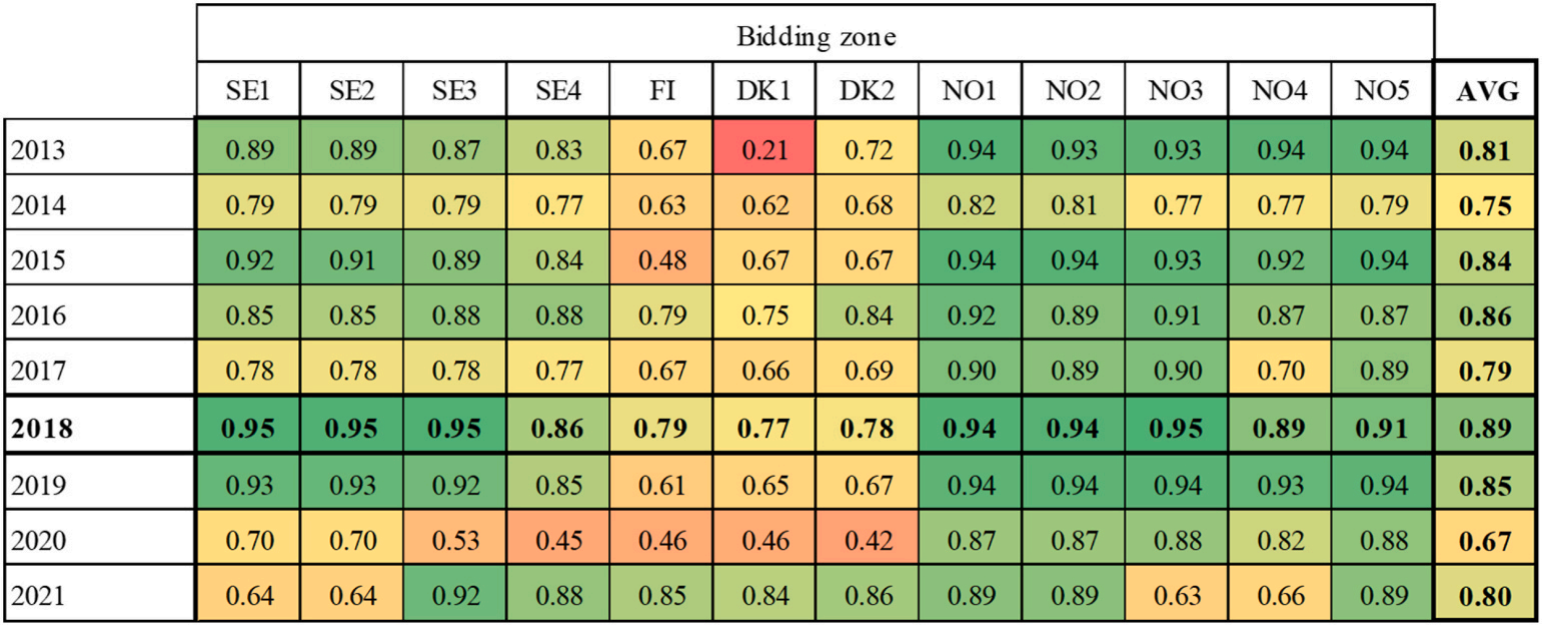
Correlation between the Nordic system price and individual bidding zone prices.

**Fig. B1:**
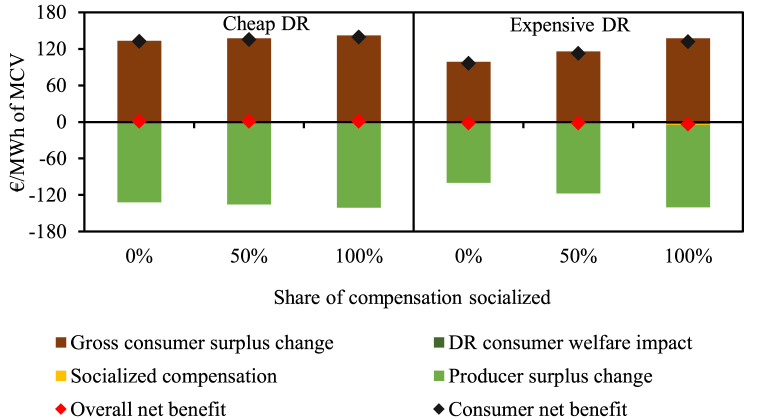
Overall net benefit components during the most expensive hour in 2018 with *Cheap DR* and *Expensive DR* activation cost curves and varied compensation socialization.

**Fig. B2:**
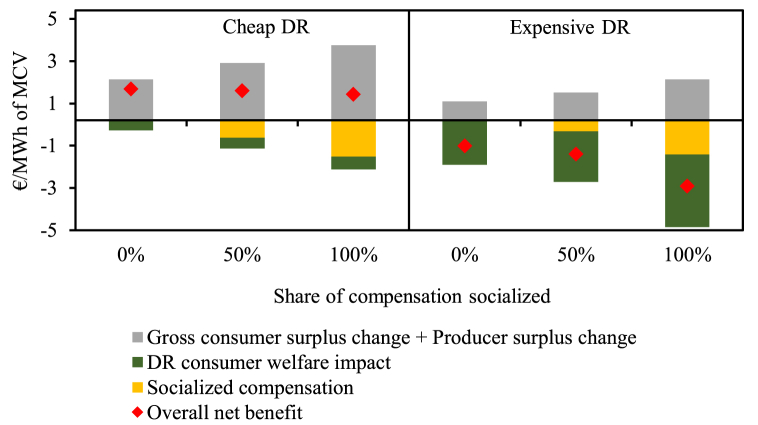
Overall net benefit components during the most expensive hour in 2018 with *Cheap DR* and *Expensive DR* activation curves and varied compensation socialization (*with gross consumer and producer surplus changes summed and adjusted to scale*).

**Fig. C1:**
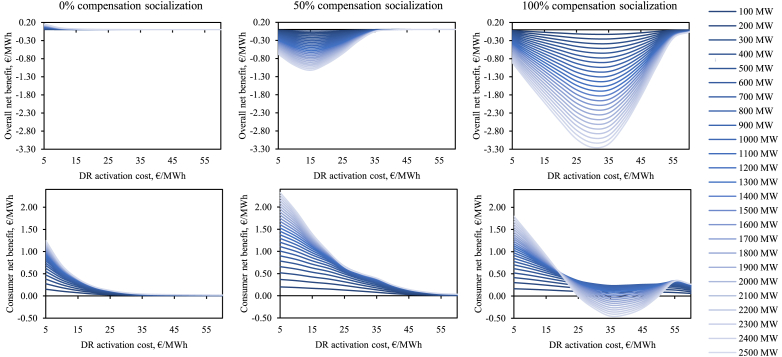
Overall net benefit and consumer net benefit as a function of DR activation cost at varied hourly load-reducing DR volumes in three compensation socialization alternatives.

## Appendix A Nordic system price correlation with DA market price in different bidding zones

To select the most suitable study year, we performed a correlation analysis between the hourly Nordic system price and the respective DA market price in different bidding zones of the Nordic countries (Fig. A1). This is necessary because for the case study we need to use the system price as a reasonable proxy for an aggregate of the area prices, since individual bidding zone market curve data is not openly available. Otherwise (i.e., if the areas are weakly correlated), it would be preferential to model the distinct bidding zone prices separately).

We observe that the system price was overall best correlated with individual bidding zone prices in the year 2018. Consequently, it was favored for the study. Moreover, at the time of writing this paper, the data published from June 2020 onwards could not be used for calculations, as after the launch of the multi-NEMO arrangement in the Nordic countries, Nord Pool cannot include in its published aggregated bid curves bids placed by Nordic market participants with other market operators (i.e., EPEX SPOT). Therefore, the published data from mid-2020 onwards is insufficient for correct replication of the Nordic system price [35]. These issues do not concern the study year of 2018.

Overall, in 2018, the Nordic system price had an average value of 43.99 €/MWh, median of 44.54 €/MWh and standard deviation of 9.94 €/MWh. The minimum and maximum price was 2.17 €/MWh and 198.29 €/MWh, respectively, however, in 3 h only the price exceeded 100 €/MWh. For 2018, there was a strong correlation (*r* > 0.75) with each bidding zone, the average coefficient being 0.89. The system price is most closely linked to the area prices for Sweden and Norway both in terms of the overall trend and specifically in 2018.

Concerning, Fig. A1, note that the extreme outlier of *r* = 0.21 for DK1 price correlation with the system price in 2013 can be explained by the presence of extraordinary supply shortage in the area during 5 h on 7 June 7, 2013 when the demand bids were subjected to pro-rata reductions in 2 h, setting the price to then maximum 2000 €/MWh [38].

## Appendix B Overall net benefit analysis during the most expensive hour

Here we investigate in more detail the welfare impacts during the hour with the largest original market price in the studied time period. It was the 1425th hour in 2018 with a system price of 198.27 €/MWh. We consider the different components of the overall net benefit as per Section 3.2. For this, in Fig. B1 we visualize the results for selected cases: the *Cheap DR* and *Expensive DR* curves and compensation socialization alternatives at 0 %, 50 % and 100 %.

Evidently, due to the very large MCP reduction achieved in all the presented alternatives, the gross consumer surplus change and producer surplus change are much larger than the other two components (socialized compensation and DR consumer welfare impact) which are barely visible in Fig. B1. To alleviate this, we can sum the two largest components and thus obtain charts with a more granular scale (Fig. B2) allowing us to analyze the results in more detail. It reiterates the peculiar observation already evident in Fig. 9: for the *Cheap DR* case the overall net benefit is positive and largely similar for the studied socialization alternatives, whereas for *Expensive DR* the net benefit is negative and even more decreasing with greater socialization. The chart allows us to make two conjectures as to why this is the case.

First, the gross consumer surplus change and producer surplus change sum is positive for both considered DR activation curves, but notably more so with *Cheap DR*. This is likely driven by the *Cheap DR* being able to achieve more significant market price reductions (by 134.28 … 143.03 €/MWh with *Cheap DR* vs by 99.28–138.27 €/MWh with *Expensive DR*). Price reduction generally induces larger gross consumer surplus increase than the corresponding producer surplus decrease.

Second, for the *Expensive DR* activation cost curve, the negative DR consumer welfare impact is significantly larger than for the *Cheap DR*. This is driven purely by the assumed shape of the respective DR activation cost curve. In the benchmarks (no exposure to market, fixed retail rate), the over-consumption is vastly greater with the *Expensive DR* (a positive value as per the top-left chart in Fig. 5). With exposure to market and 0 % of the compensation socialized, the DR consumers are at their efficient consumption, and there is no over-consumption (this creates a negative DR consumer welfare impact as per ${\Delta DRW}_{t,b,s}^{\text{alt}}-{\Delta DRW}_{t,b}^{\text{bm}}$). When the compensation payment is socialized, the negative welfare impact of losing the over-consumption is further amplified by under-consumption induced by the socialization (bottom-left of Fig. 5), which in this instance is also notably higher for the *Expensive DR* case, as seen in Fig. B2.

## Appendix C Sensitivity analysis of DR activation cost and volume impact

In order to assess with more nuance the impact of load-reducing DR activation cost and volume on the socioeconomic benefits obtainable from IA participation in the DA market, we repeat the simulations with simple single-step bids. Unlike the results presented in the body of the paper where three multi-step DR cost curves where utilized, here we assume that the IAs submit a single price-quantity pair. The DR activation cost is varied from 5 to 60 €/MWh with a step of 5 €/MWh (mimicking the price steps of the previously used curves). The hourly volume of DR bid is varied from 100 to 2500 MW, with a step of 100 MW. The results for three compensation socialization alternatives are summarized in Fig. C1, where each blue line corresponds to one DR volume assumption (darker colors correspond to smaller volumes, and lighter shades to larger ones).

The overall net benefit results (top graphs) in principle conform to the previously assessed multi-step scenarios, showing generally negative benefit, except for the no socialization alternative. However, the sensitivity analysis exposes the impact of DR activation cost at high marketed volumes. In particular, it is evident that in the specific year studied, the most harmful to the overall benefit were DR resources with activation cost in the medium range (for full socialization cases) and in the medium-low range (for the 50 % socialization case). If no socialization is envisioned, load-reducing DR in the DA market via IAs poses no negative overall net benefit risks, regardless of the bid volume.

On the other hand, the consumer net benefit metric (bottom graphs) shows that the most benefit can be achieved with cheap DR resources. Furthermore, as their activation cost increases, the consumer net benefit quickly reduces. A strong self-cannibalizing effect can be also observed: the increment in consumer net benefit growth decreases with each higher step in DR bid volume (discernible as a reduced vertical distance between every two adjacent lines). Particularly strongly the over-incentivization of load-reducing DR is evident in the bottom-right chart of Fig. C1, where at 100 % socialization any bid volume above 1600 MW produces increasingly negative consumer net benefit in the medium activation cost range.

## Footnotes

6 The available datasets only contain information about the total volumes of cleared block orders and the net flows with neighboring bidding areas in each hour, thus changes in these parameters cannot be modeled with the available data, and a simplification is necessary.

7 Which is about 6 % of the average hourly DA volume in 2018 (42 GWh).

## Footnotes

1 In that regard, IAs in the United States do not have to pay a compensation if a so-called “net benefits test” is passed [39].

2 Furthermore, in terms of IA direct participation in the day-ahead market, NordReg proposes utilizing a split-supply model, whereby the IAs act as suppliers for the flexible consumption devices. However, to comply with the Clean Energy Package requirements, also with the split-supply model in place a regulatory framework has to be created on top of it in order to enable independent aggregation [14]. In that regard, a study commissioned by the Swedish Energy Markets Inspectorate [40] argues that also separate centralized settlement model should be devised, preferably with the retail rate as the compensation price.

3 The volumes of energy transferred by an IA can be expected higher in the DA wholesale market compared to reserve markets, in which the main renumeration comes from capacity and the activations are relatively short in duration. Thereby the IA-supplier compensation issue is more pressing in the DA market.

4 Since the launch of the Nordic multi-NEMO arrangement in June 2020, electricity wholesale in the Nordic region can be organized by various market operators and is subject to their competition. However, this has negatively impacted the data availability on system-wide market clearing results.

5 As a simplification we assume that the compensation payment is paid as a lump sum and does not impact the retail rate. Moreover, the treatment of load-increasing DR compensation being exactly inverse to the load-reducing DR is necessary to ensure consistency of the model. In practice, special treatment of load-increasing DR might be also feasible, as it is generally less beneficial to the system at large. Under the current assumptions, a full compensation socialization effectively disqualifies load-increasing DR, as IAs are not expected to engage in activities that could bring them loss.

## References

[1] Holttinen H., Tuohy A., Milligan M., Lannoye E., Silva V., Muller S. (2013). The flexibility workout: managing variable resources and assessing the need for power system modification. IEEE Power Energy Mag..

[2] European Commission (2016). https://energy.ec.europa.eu/publications/impact-assessment-study-downstream-flexibility-price-flexibility-demand-response-smart-metering_en.

[3] SmartEn EU market monitor for demand-side flexibility 2020 2021:32. https://smarten.eu/eu-market-monitor-for-demand-side-flexibility-2020/.

[4] Gils H.C. (2014). Assessment of the theoretical demand response potential in Europe. Energy.

[5] Schittekatte T., Reif V., Meeus L. (2021). Welcoming new entrants into European electricity markets. Energies.

[6] Poplavskaya K., de Vries L., Sioshansi F.P. (2020). Behind beyond M..

[7] Reif V., Nouicer A., Schittekatte T., Deschamps V., Meeus L. (2021). D9.12 Report on the Foundations for the adoptions of New Network Codes 1.

[8] Directive (EU) (2019).

[9] de Heer H., van der Laan M. (2017). https://www.usef.energy/app/uploads/2017/09/Recommended-practices-for-DR-market-design-2.pdf.

[10] Schittekatte T., Deschamps V., Meeus L. (2021). The regulatory framework for independent aggregators. Electr. J..

[11] Pöyry Management Consulting Independent aggregator models. Final report 2018:43. https://tem.fi/documents/1410877/3481825/Itsenäisen+aggregaattorin+mallit+26.6.2018/f63589df-49ea-4232-b39a-bb6973407fe2/Itsenäisen+aggregaattorin+mallit+26.6.2018.pdf.

[12] Sadovica L., Marcina K., Lavrinovics V., Junghans G. (2017). 2017 IEEE 58th Int. Sci. Conf. Power Electr. Eng. Riga Tech. Univ..

[13] Chua-Liang Su, Kirschen D. (2009). Quantifying the effect of demand response on electricity markets. IEEE Trans. Power Syst..

[14] NordREG Nordic regulatory framework for independent aggregation 2020:43. http://www.nordicenergyregulators.org/wp-content/uploads/2021/05/A-New-Regulatory-Framework_for_Independent_Aggregation_NordREG_recommendations_2020_02.pdf.

[15] (2024). EU DSO Entity and ENTSO-E Proposal for a Network Code on Demand Response.

[16] ACER (2023). Demand response and other distributed energy resources: what barriers are holding them back? 2023 Market Monitoring Report. https://www.acer.europa.eu/sites/default/files/documents/Publications/ACER_MMR_2023_Barriers_to_demand_response.pdf.

[17] Walawalkar R., Blumsack S., Apt J., Fernands S. (2008). An economic welfare analysis of demand response in the PJM electricity market. Energy Pol..

[18] Chao H. (2011). Demand response in wholesale electricity markets: the choice of customer baseline. J. Regul. Econ..

[19] Cicala S. (2022). Imperfect markets versus imperfect regulation in US electricity generation. Am. Econ. Rev..

[20] Ye Y., Papadaskalopoulos D., Strbac G. (2018). Investigating the ability of demand shifting to mitigate electricity producers' market power. IEEE Trans. Power Syst..

[21] Nord Pool. System Price Curve Data n.d https://www.nordpoolgroup.com/en/elspot-price-curves/(accessed February 6, 2024).

[22] Arcos-Vargas A., Nuñez F., Román-Collado R. (2020). Short-term effects of PV integration on global welfare and CO2 emissions. An application to the Iberian electricity market. Energy.

[23] Roldán-Fernández J.M., Burgos-Payán M., Riquelme-Santos J.M. (2021). Impact of domestic PV systems in the day-ahead Iberian electricity market. Sol. Energy.

[24] Roldán Fernández J.M., Payán M.B., Santos J.M.R., García Á.L.T. (2017). The voluntary price for the small consumer: real-time pricing in Spain. Energy Pol..

[25] Roldán Fernández J.M., Burgos Payán M., Riquelme Santos J.M., Trigo García Á.L. (2016). Renewable generation versus demand-side management. A comparison for the Spanish market. Energy Pol..

[26] Ciarreta A., Espinosa M.P., Pizarro-Irizar C. (2014). Is green energy expensive? Empirical evidence from the Spanish electricity market. Energy Pol..

[27] Baltputnis K., Broka Z. (2021). Estimating the benefit from independent aggregation in the day-ahead market. Latv. J. Phys. Tech. Sci..

[28] Barbero M., Corchero C., Canals Casals L., Igualada L., Heredia F.-J. (2020). Critical evaluation of European balancing markets to enable the participation of Demand Aggregators. Appl. Energy.

[29] Akkouch R., Menci S.P., Pavic I. (2024). 2024 20th Int. Conf. Eur. Energy Mark..

[30] Bredesen H.-A. (2016).

[31] Chen Y., Hexeberg A., Rosendahl K.E., Bolkesjø T.F. (2021). Long‐term trends of Nordic power market: a review. WIREs Energy Environ.

[32] The Swedish Energy Markets Inspectorate (2020). The Swedish electricity and natural gas market 2019. https://ei.se/download/18.5b0e2a2a176843ef8f5af7eb/1612525440725/The-Swedish-electricity-and-natural-gas-market2019-Ei-R2020-07.pdf.

[33] Hu X., Jaraitė J., Kažukauskas A. (2021). The effects of wind power on electricity markets: a case study of the Swedish intraday market. Energy Econ..

[34] ACER, CEER Annual report on the results of monitoring the internal electricity and natural gas markets in 2019 2020. https://acer.europa.eu/sites/default/files/documents/Publications/ACERMarketMonitoringReport2019-ElectricityWholesaleMarketsVolume.pdf.

[35] Nord Pool (2020). How to interpret the information in market cross point data reports–Nordic system price curves. https://www.nordpoolgroup.com/4a67db/globalassets/information-in-market-cross-point-data-reports.pdf.

[36] Schwartz D. (2017). https://se.mathworks.com/matlabcentral/fileexchange/11837-fast-and-robust-curve-intersections.

[37] Nord Pool AS. Nordic System Price (2022). https://www.nordpoolgroup.com/4ac5b1/globalassets/download-center/day-ahead/methodology-for-calculating-nordic-system-price---november-2022.pdf.

[38] Nord Pool AS Undersupply leads to pro-rata reduction of DK1 purchase orders for 7 June 2013 2013. https://www.nordpoolgroup.com/en/trading/Operational-Message-List/2013/06/1/.

[39] Panfil M. (2015). Demand response, order 745 and the supreme court. Electr. J..

[40] Dnv G.L., Sweden A.S. (2020). Impact assessment of different models of independent aggregator financial responsibility and compensation in Sweden. https://ei.se/download/18.22acd6711784a1f3a5b1790/1616425577180/Oberoende%20aggregatorer_DNVGL%20Slutrapport.pdf.

